# Re-examination of the *APETALA2/Ethylene-Responsive Factor* Gene Family in Barley (*Hordeum vulgare* L.) Indicates a Role in the Regulation of Starch Synthesis

**DOI:** 10.3389/fpls.2021.791584

**Published:** 2021-12-01

**Authors:** Jinjin Ding, Hassan Karim, Yulong Li, Wendy Harwood, Carlos Guzmán, Na Lin, Qiang Xu, Yazhou Zhang, Huaping Tang, Yunfeng Jiang, Pengfei Qi, Mei Deng, Jian Ma, Jirui Wang, Guoyue Chen, Xiujin Lan, Yuming Wei, Youliang Zheng, Qiantao Jiang

**Affiliations:** ^1^State Key Laboratory of Crop Gene Exploration and Utilization in Southwest China, Sichuan Agricultural University, Chengdu, China; ^2^Triticeae Research Institute, Sichuan Agricultural University, Chengdu, China; ^3^John Innes Center, Norwich Research Park, Norwich, United Kingdom; ^4^Departamento de Genética, Escuela Técnica Superior de Ingeniería Agronómica y de Montes, Edificio Gregor Mendel, Campus de Rabanales, Universidad de Córdoba, Córdoba, Spain; ^5^College of Sichuan Tea, Yibin University, Yibin, China

**Keywords:** barley, *APETALA2/Ethylene-Responsive*, transcription factors, gene interaction, starch synthesis

## Abstract

The *APETALA2/Ethylene-Responsive* factor (*AP2/ERF*) gene family is a large plant-specific transcription factor family, which plays important roles in regulating plant growth and development. A role in starch synthesis is among the multiple functions of this family of transcription factors. Barley (*Hordeum vulgare* L.) is one of the most important cereals for starch production. However, there are limited data on the contribution of AP2 transcription factors in barley. In this study, we used the recently published barley genome database (Morex) to identify 185 genes of the *HvAP2/ERF* family. Compared with previous work, we identified 64 new genes in the *HvAP2/ERF* gene family and corrected some previously misannotated and duplicated genes. After phylogenetic analysis, *HvAP2/ERF* genes were classified into four subfamilies and 18 subgroups. Expression profiling showed different patterns of spatial and temporal expression for *HvAP2/ERF* genes. Most of the 12 *HvAP2/ERF* genes analyzed using quantitative reverse transcription–polymerase chain reaction had similar expression patterns when compared with those of starch synthase genes in barley, except for *HvAP2-18* and *HvERF-73*. *HvAP2-18* is homologous to *OsRSR1*, which negatively regulates the synthesis of rice starch. Luciferase reporter gene, and yeast one-hybrid assays showed that *HvAP2-18* bound the promoter of *AGP-S* and *SBE1 in vitro*. Thus, *HvAP2-18* might be an interesting candidate gene to further explore the mechanisms involved in the regulation of starch synthesis in barley.

## Introduction

Transcription factors (TFs) bind to the cis-acting elements of their target genes and play a key role in gene transcription regulation. The *APETALA2/Ethylene-Responsive Factor* (*AP2/ERF*) superfamily includes the *AP2* (APETALA2), *ERF* (ethylene-responsive factors), and *RAV* (related to ABI3/VP) gene families and is one of the largest groups of TFs in plants. TFs of the AP2/ERF superfamily contain an AP2 DNA-binding domain (Riechmann and Meyerowitz, 1998; Sakuma et al., 2002). Additionally, the *AP2/ERF* superfamily is defined by the AP2/ERF domain, which comprises approximately 60–70 amino acids and is involved in DNA-binding (Kumar et al., 2009). The *AP2* subfamily members contain two AP2/ERF domains lacking a conserved WLG motif. The *ERF* subfamily possesses only one AP2/ERF domain, and the RAV subfamily members have a single AP2/ERF domain and a B3 domain (Sakuma et al., 2002). The *ERF* family is further split up into two subfamilies according to the DNA sequence bound: ERF and CBF/DREB (Sakuma et al., 2002). Proteins encoded by genes from the *ERF* subfamily bind to the core motif AGCCGCC (Zhou et al., 1997), whereas the CBF/DREB subfamily contains C-repeats recognizing the cis-acting element, A/GCCGAC (Yamaguchi-Shinozaki and Shinozaki, 1994).

The AP2 domain was first described in Arabidopsis and is involved in flower development (Jofuku et al., 1994). AP2/ERF proteins have important functions in the transcriptional regulation of various biological processes related to growth and development, as well as various responses to environmental stimuli (Moose and Sisco, 1996; Liu et al., 2013). Indeed, the combined use of genetic and molecular approaches has shown that the *AP2/ERF* family participate in the regulation of developmental processes, such as flower development (Elliott et al., 1996), spikelet meristem determinacy (Chuck et al., 1998), leaf epidermal cell identity (Moose and Sisco, 1996), and embryo development (Boutilier et al., 2002). Extensive plant genome sequencing has identified the *AP2/ERF* gene family in various plants, such as Arabidopsis (Moose and Sisco, 1996), rice, maize (Liu et al., 2013), soybean (Zhang et al., 2008), and foxtail millet (Lata et al., 2014). Although there are data available on the *AP2/ERF* family in barley (Hordeum vulgare L) (Guo et al., 2016), no investigation has been conducted based on the latest available genomic database.

Barley is the fourth most abundant cereal, after rice, wheat, and corn, in both area and tonnage harvested. It is widely used for feeding animals and beer making (Mayer et al., 2012). The International Barley Sequencing Consortium (IBSC) released the genome sequencing map of barley cultivar Morex, a North American spring six-row malting barley, for the first time in 2012 (Mayer et al., 2012). This map is referred later in the text as V1. In 2017, hierarchical shotgun sequencing of bacterial artificial chromosomes was combined with the use of optical mapping and chromosome-scale scaffolding with chromosome conformation capture sequencing (Hi-C) by the IBSC to create a highly contiguous reference genome sequence for Morex (Mascher et al., 2017). The reference genome assembly for Morex was improved by Monat et al. (2019) with the use of TRITEX, an open-source computational workflow and is referred later in the text as Morex V2. It represents a significant resource for the barley research community. The improved barley genome provides a good opportunity to make better use of barley germplasm resources and carry out the cloning and functional characterization of unknown genes. Specifically, the second version of the reference genome provided the basis for the re-analysis of the *HvAP2/ERF* gene family in barley.

Starch, the most abundant component of cereal grains, has important biological functions and is a major part of the human diet (Sonnewald and Kossmann, 2013). Starch content and composition are key elements influencing grain yield and quality. They play important roles during endosperm development (James et al., 2003). Starch consists of two types of glucose polymers, namely, amylose and amylopectin (Nakamura et al., 1995). Starch biosynthesis can be broadly divided into three stages: sucrose transport, synthesis of the glucosyl donor, and amylopectin or amylose synthesis (Keeling and Myers, 2010; Bahaji et al., 2014). Starch synthesis in cereals requires several well-characterized enzymes, including ADP-glucose pyrophosphorylase (AGPase), granule bound starch synthase (GBSS), starch synthase (SS), starch branching enzyme (Sun et al., 2003), and starch debranching enzyme (DBE) (James et al., 2003; Hannah and James, 2008; Bahaji et al., 2014). ADP-glucose enters the amyloplast through Brittle1 (BT1, the transporter of ADP-glucose) to be used as a substrate for starch biosynthesis (Bahaji et al., 2014). GBSS is responsible for amylose synthesis and mutation of this enzyme results in lower amylose content (Pérez et al., 2019). Amylopectin synthesis is a complex process involving interaction and feedback between the enzymes SS, SBE, and DBE (Hannah and James, 2008; Jeon et al., 2010).

TFs play an important role in the regulation of starch synthesis. Indeed, previous studies identified TFs regulating starch synthesis in rice, maize, wheat, and, to a lesser extent, barley. In rice, TF genes such as *NF-YB1*, *NF-YC12*, *OsbZIP58*, *OsbZIP76*, and *Rice Starch Regulator 1* (*RSR1*) have been shown to regulate starch synthesis (Fu and Xue, 2010; Wang et al., 2013; Bai et al., 2016; Bello et al., 2019; Niu et al., 2020). *ZmNAC36*, *ZmbZIP91, ZmbZIP22, Opaque 2, Opaque 11, ZmEREB156, ZmNAC128*, and *ZmNAC130* are central players regulating the expression of starch biosynthesis genes in maize (Zhang et al., 2014, 2016, 2019; Chen et al., 2016; Huang et al., 2016; Feng et al., 2018; Dong et al., 2019). In wheat, *TaRSR1*, a gene homologous to *OsRSR1* and *TaNAC019-A1*, negatively regulates the expression of many starch synthesis genes, and *TubZIP28* and *TabZIP28* are transcriptional activators of starch synthesis (Liu et al., 2014, 2016; Song et al., 2020). *SUSIBA2*, a member of the WRKY TF family, was identified in rice, maize, wheat, and barley (Sun et al., 2003).

The accurate analysis of the *AP2/ERF* gene family members is important for screening starch synthesis-related genes. In this study, we used the latest published barley genome database for the identification and classification of *HvAP2/ERF* family members. The data were also confirmed using the Golden Promise genome database. Furthermore, to confirm our results, we compared the latest published database with the previous version. A comprehensive analysis of structural features, phylogenetic relationships, chromosomal location, and expression patterns of the identified *AP2/ERF* family members was performed. Data regarding the expression of *AP2* family members in different barley tissues were retrieved from the database, and the co-expression of these *AP2* family members with starch synthesis genes was analyzed. Our results provide new insight into the link between the *AP2/ERF* family and starch synthesis genes. Particularly, we identified *HvAP2-18* as a candidate gene binding to the promoter of *AGP-S* and *SBEI* to suppress starch synthesis. The identification of other candidate genes might also provide a better understanding of the *AP2* family contribution to starch synthesis in barley.

## Materials and Methods

### *HvAP2/ERF* Gene Family Sequence Database Searches

We used two methods to comprehensively identify AP2/ERF domain-containing sequences in barley. The first method involved retrieving the *HvAP2/ERF* gene family members from the Morex genome on IPK^1^ by using the keyword “AP2/ERF” as input (in Morex v2 Gene Models-2019). The second method consisted in downloading genome sequences including DNA fasta and GFF3 files from the e!DAL database.^2^ The PlantTFDB v5.0 database^3^ was used to download the protein sequences of *HvAP2/ERF* TFs from *Hordeum vulgare* and *Arabidopsis thaliana*. These were then used for the first BLAST search (*e*-value ≤ 1e-10) in the Morex genomes, using the TBtools (Chen et al., 2020). Redundant sequences were manually removed. The data extracted from this first BLAST search were used as a query for a second BLAST search (*e*-value ≤ 1e-10). We compared the results, downloaded them, and manually removed non-AP2/ERF members. The sequences of the proteins identified from the two BLAST searches were further analyzed for the presence of the conserved AP2/ERF domain, using the NCBI Conserved Domain Database server^4^ (Marchler-Bauer et al., 2010). The proteins in which the presence of the AP2/ERF domain was confirmed were considered as putative *AP2/ERF* TFs.

### Identification and Comparison of *HvAP2/ERF* Gene Family From the New and Old Barley Genome Versions

The sequences of *HvAP2/ERF* genes were searched and downloaded from the Molex WGS Gene Models (2012) in the IPK database^5^ in a previous study (Guo et al., 2016). We used sequences of the *HvAP2/ERF* genes from this previous study as queries to do a BLAST search in the Morex V2 (all Morex V2 in this article refer to the barley genome published in 2019) database to allow a comparison of our results for these studies. The gene with the Expect = 0 value was the same gene with the query sequence. Next, the *HvAP2/ERF* family genes identified from the Morex V2 genome were used for BLAST searches (*e*-value ≤ 1e-10) in the IPK HC_genes_CDS_Seq_2012, LC_genes_CDS_Seq_2012 and full-length cDNA databases.^6^ The *HvAP2/ERF* genes identified in the different versions of the barley genome could be analyzed through the two forward and reverse BLASTs. DNAMAN was used for multiple alignments of uncertain genes. The genes differentially identified in previous studies and the present work were used for BLAST search in the Golden Promise genome.^7^

### Gene Structure and Phylogenetic Analysis

The coding sequence of each *HvAP2/ERF* gene was aligned with its genomic sequence using TBtools to construct an exon/intron map. To identify the evolutionary relationships between AP2/ERF proteins from barley and *Arabidopsis thaliana*, all the amino acid sequences were aligned using the ClustalW program implemented in MEGAX.^8^ The phylogenetic tree was constructed using the neighbor-joining method based on the JTT matrix-based model with 1,000 bootstrap replications.

### Conserved Motif Analysis and Localization of *HvAP2/ERF* Genes on Morex Chromosomes

The online software MEME 5.1.1^9^ was used to search the AP2/ERF protein sequence motifs, with the following parameters: number of repetitions, any; maximum number of motifs, 20; and optimum motif width, ≥ 6 and ≤ 200 (Ma et al., 2017). All *HvAP2/ERF* genes identified were analyzed by mapping the sequences back to the corresponding genome annotation GFF3 file using TBtools to obtain the chromosomal locations.

### Expression Analysis

All the *HvAP2/ERF* gene coding DNA sequence (CDS) were compared with the transcriptomic database in the Barley Reference Transcript (BaRTv1.0) Dataset^10^ (Rapazote-Flores et al., 2019). The RNA-seq data of 13 tissues [Root (10 cm seedlings), Root 2 (4-week-old seedlings), Shoot (10 cm seedlings), Rachis (5 weeks postanthesis), Senescing leaf (2 months), Tillers (3rd internode), Inflorescence-1 (0.5 cm)], Inflorescence-2 [([1–1.5 cm], Embryo (germinating), Palea (6 weeks pa), Epidermis 4 weeks), grain (5 days postanthesis DPA), and grain (15 DPA)] of Morex were retrieved from the James Hutton Institute,^11^ and the log2 of the transcripts per million value for each *HvAP2/ERF* gene was visualized as a heat map with a blue–yellow–red gradient.

### Plant Growth, RNA Extraction, and Quantitative Reverse Transcription–Polymerase Chain Reaction Analysis

Barley key starch synthase genes and 12 *HvAP2/ERF* genes with high expression levels were quantified to verify the RNA-seq data and screen for *HvAP2/ERF* candidate genes. Barley accession “Golden Promise” was grown in a phytotron chamber under 16 h light/8 h dark and 24°C day/18°C night temperature cycles. The grains were harvested at 5, 10, 15, 20, and 25 DPA, transferred promptly into liquid nitrogen, and stored at -80°C until RNA extraction. Total RNA was isolated using the Plant RNA kits (Biofit, Chengdu, China) according to the manufacturer’s instructions. Each developmental stage was prepared and tested in three biological replicates. First-strand cDNAs were synthesized using PrimeScript^TM^ RT reagent kits with gDNA Eraser (TaKaRa, Dalian, China). The quantitative reverse transcription–polymerase chain reaction (qRT-PCR) was carried out with SYBR^®^ Premix Ex Taq^TM^ II (TaKaRa) on a CFX 96 Real-Time System (Bio-Rad, Hercules, United States). The CFX Manager software (Bio-Rad, Hercules, United States) was used to analyze the qRT-PCR data and to calculate the relative expression using the 2^–△△^ Ct method. The barley β-actin and glyceraldehyde 3-phosphate dehydrogenase genes were used as internal reference genes to normalize the relative expression of the candidate genes.

### Dual-Luciferase Reporter Assay

A 1.5 kb portion of the promoter sequence from either *HvAGP-S*, *HvAGP-L*, *SS2a*, *Waxy*, *SBE2a*, *SBE1*, or *SS1* was cloned into the pGreenII 0800-Luc vector to create the promoter–reporter controlling the firefly luciferase construct. The *HvAP2-18* CDS was cloned into the pGreenII 62-SK vector to create pGreenII 62-SK-*HvAP2-18*, which was used as the effector vector. Both vectors were co-expressed in Tobacco. Cotransfected Tobacco was cultured overnight in the dark. Luciferase activities were measured using the dual-luciferase reporter (LUC) assay kit (Yeasen) and the GLOMAX 20/20 Luminometer (Promega Madison, WI, United States).

### Yeast One-Hybrid Assay

The yeast one-hybrid (Y1H) assay was conducted following the protocol of the Matchmaker Gold Yeast One-Hybrid Library Screening System (Clontech, Palo Alto, CA, United States). The promoters of *HvAGP-S*, *HvSBE1*, and *HvSS2a* were subcloned into the pAbAi vector to produce a bait construct. The construct was linearized by digestion with *Bst*BI and integrated into the URA3–52 locus of the Y1HGold yeast genome to generate a Y1H bait strain. The coding sequences of *HvAP2-18* were cloned into the pGADT7 vector to generate the pGADT7-TFs construct. These constructs or the empty vector were separately transformed into the Y1H bait strain and selected on a synthetic dropout (Schmidt et al., 2013)/-Leu plate containing 100 ng/mL aureobasidin A.

## Results

### Identification of the *HvAP2/ERF* in Morex

The updated version Morex V2 of the barley genome database (Monat et al., 2019) is more accurate for the characterization of *HvAP2/ERF* family members than the previous version. We identified a total of 185 non-redundant *HvAP2/ERF* genes in Morex via genome-wide search (Supplementary Table 1). These genes were further divided into four groups according to the characteristics of each AP2/ERF subfamily. The *AP2* subfamily, which has two AP2 domains and no WLG motif, contained 33 genes (Supplementary Figure 1). The DREB and ERF subfamilies, which have one AP2 domain, had 58 and 85 genes, respectively. The RAV family, which is characterized by an AP2 domain and a B3 domain, contained nine genes. All the identified *HvAP2/ERF* genes encoded proteins with lengths ranging from 85 (HvERF41) to 700 (HvAP2-6) amino acids, protein mass between 13.38 and 69.73 kD, and protein pI ranging from 3.98 (HvDREB2.11) to 11.87 (HvERF6.5).

### Comparison of *HvAP2/ERF* Genes Identified in the Different Versions of the Barley Genome

There are a few published studies that used the old version of the barley genome for the identification of *HvAP2/ERF* genes. The results of the present work were compared with each of these studies. Guo et al. (2016) found 121 genes belonging to the *HvAP2/ERF* family using the old version of the barley genome. We have identified 84 newly annotated genes in the *HvAP2/ERF* family, which were also found in the Golden Promise genome by BLAST (Supplementary Table 2). The newly discovered TFs belong to different subgroups, and a maximum of 39 TFs were from the *ERF* subfamily, whereas 17, 23, and 3 were found in the *AP2, DREB*, and *RAV* families, respectively. Although *HvERF3*, *HvERF4*, and *HvERF7* contained an AP2 domain, they were less than 90% matched in the Golden Promise genome. Additionally, the previously identified *HvERF3.1* and *HvERF3.2* were identified as *HvERF-16* in the Morex V2 genome. Similarly, *HvERF4.9* and *HvERF4.10* were identified as *HvERF27*, and *HvERF2.15*, *HvERF2.16*, and *HvERF2.17* were newly annotated as *HvERF-58*. Multiple sequence alignments showed that this phenomenon might have been caused by incorrect splicing or misannotation of the 2012 genome version. We identified a larger number of *HvAP2/ERF* genes from the Morex V2 dataset than found in previous studies, leading to a more complete and accurate description of this important gene family.

### Phylogenetic Analysis of *HvAP2/ERF* Genes

**FIGURE 1:**
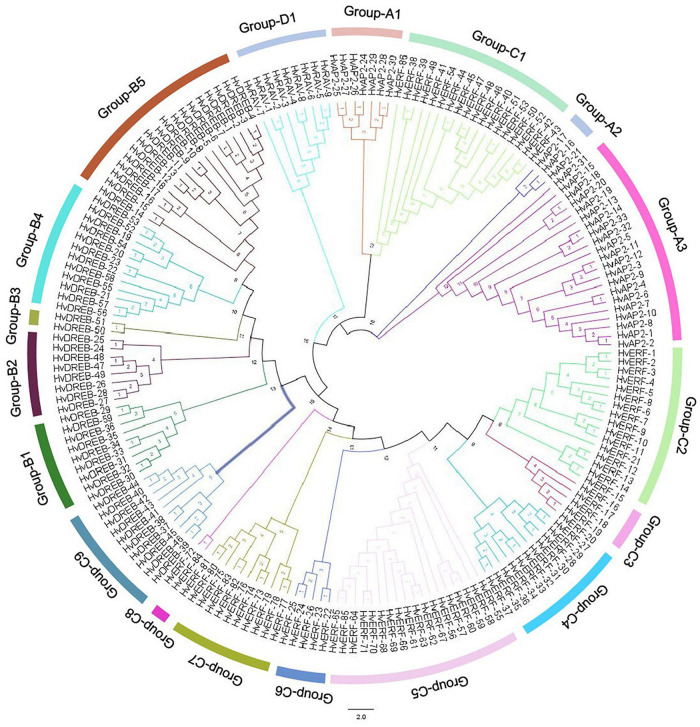
Phylogenetic classification of barley HvAP2/ERF proteins. The 18 classes are represented by branches of different colors.

The AP2/ERF family is a unique and plant-specific TF family, which significantly contributes to plant growth and development. We performed a phylogenetic analysis of all 185 *HvAP2/ERF* genes to further classify the *HvAP2/ERF* family. Consequently, we divided the AP2/ERF family into four subfamilies, which each contained subgroups. The *AP2* subfamily was formed of three subgroups (A1, A2, and A3) with 21, 3, and 7 genes, respectively. The *DREB* subfamily contained the B1–B5 subgroups, which had 18, 12, 2, 8, and 9 genes, respectively. Finally, the C1–C9 subgroups formed of 18, 16, 5, 11, 18, 5, 2, 11, and 10 genes, respectively, constituted the *ERF* subfamily, whereas the *RAV* family contained a single group, the group D1, which included nine genes. The present phylogenetic analysis is more comprehensive than that in previously published work and explains the relationship between all members of the *HvAP2/ERF* family (Figure 1).

### Structural Characteristics of the HVAP2/ERF Protein Family

**FIGURE 2:**
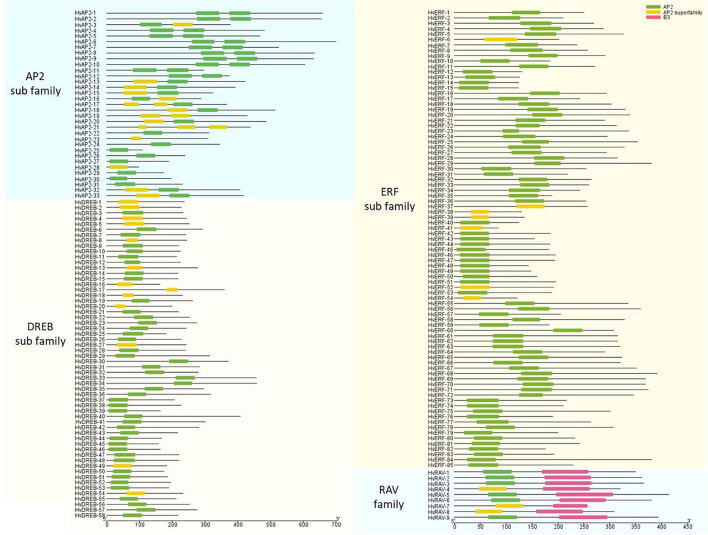
AP2 domains of barley HvAP2/ERF proteins.

**FIGURE 3:**
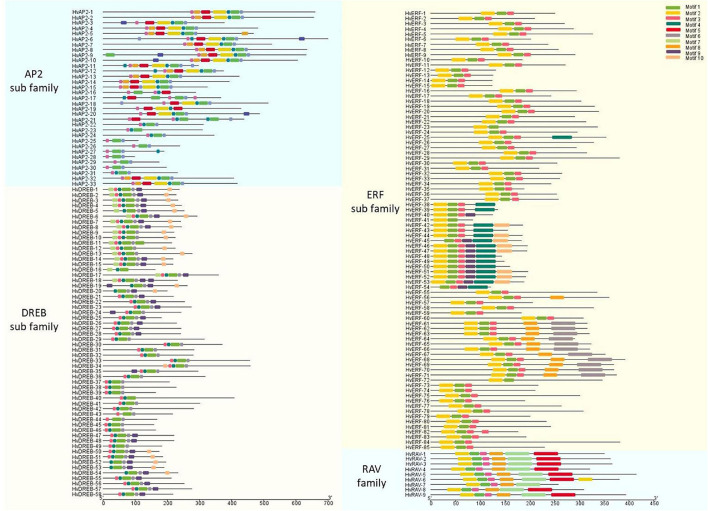
Protein motifs of barley HvAP2/ERF proteins. Conserved motifs in 185 barley HvAP2/ERF proteins. Each subfamily is represented by a different colored box.

The structural characteristics of the genes and proteins were described according to their protein domains and conserved motifs (Figures 2, 3). The MEME software was used to find conserved motifs and allowed the identification of 10 motifs in the *HvAP2/ERF* subfamilies (Supplementary Figure 2). Particularly, the MEME motif analysis revealed that different HvAP2/ERF proteins had different conserved motifs. A full motif 1 (AP2/ERF domain) was found in all HvAP2/ERF proteins. The *DREB* subfamily and some AP2 subfamilies had no motif 2, the RAV family contained motif 7, which is the B3 domain. Moreover, *HvAP2-17* and *HvAP2-21* had three AP2 domains, whereas some of the *AP2* subfamily members had only one AP2 motif lacking the WLG. Gene structural analysis showed that most of the *HvAP2/ERF* genes possessed only one exon (133/185, 71.8%). However, some genes contained more than one exon and were mostly members of the *AP2* subfamily. Supplementary Figure 3 shows that 2, 3, 4, 5, 7, 8, 9, and 10 exons were present in 32, 2, 1, 1, 4, 5, 6, and 2 genes, respectively.

### Chromosomal Distribution of *HvAP2/ERF* Genes in Morex

**FIGURE 4:**
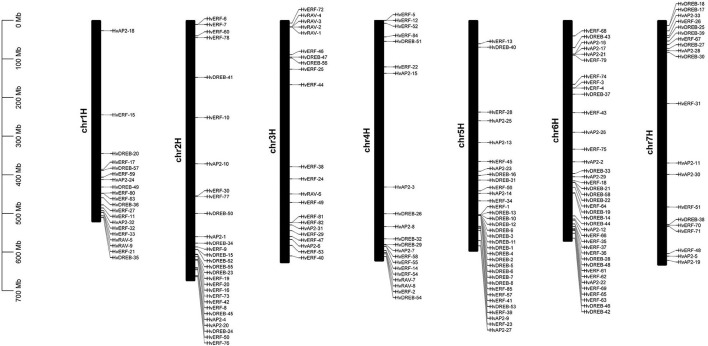
Chromosome distributions of *HvAP2/ERF* genes in barley.

On the basis of the gene annotation information, the position of 185 *HvAP2/ERF* genes on Morex chromosomes was determined. All identified genes were distributed across the whole genome (Figure 4). However, a large number of *HvAP2/ERF* family members were found clustered on chromosomes 2, 5, and 6. Additionally, genes of the *HvAP2/ERF* family were localized on chromosomes 3 and 7. The *HvAP2/ERF* family members were not evenly distributed across the genome. Most of them were located on the distal regions of chromosomes, suggesting diverse functions of *HvAP2/ERF* family members, and that interactions between them may play a role in plants.

### Expression Profiles of *HvAP2/ERF* Genes During Vegetative and Reproductive Development

**FIGURE 5:**
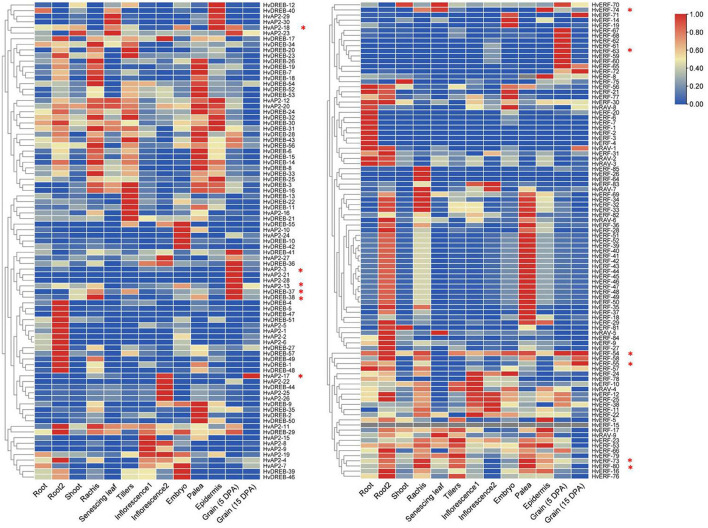
Heatmaps of expression profiles for *HvAP2/ERF* genes at different developmental stages for eight tissues in barley. The color scale represents the expression values. * Indicates the 12 genes selected for qRT-PCR verification. Root (10 cm seedlings), Root 2 (4 weeks seedling), Shoot (10 cm seedlings), Rachis (5 weeks pa), Senescing leaf (2 months), Tillers (third internode), Inflorescence-1 (0.5 cm), Inflorescence-2 (1–1.5 cm), Embryo (germinating), Palea (6 weeks pa), Epidermis (4 weeks), Grain (5 DPA) and Grain (15 DPA), DPA: days postanthesis, pa: postanthes.

**FIGURE 6:**
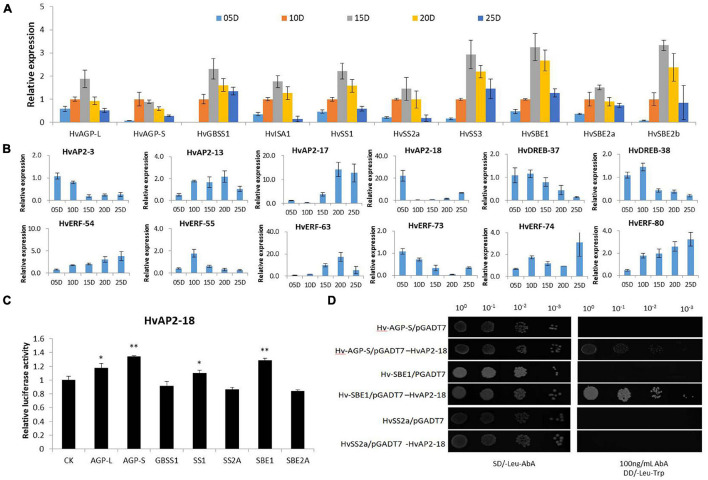
Co-expression and interaction analyses of *HvAP2/ERF* candidate genes involved in starch synthesis. **(A)** Relative expression levels of key starch synthase genes. DPA: days post anthesis. **(B)** Relative expression levels of 12 *HvAP2/ERF* genes. **(C)** Characterization of the interaction between the *HvAP2-18* protein and the promoter of starch synthase genes via LUC assay. **(D)** Characterization of the interaction between the *HvAP2-18* protein and the promoter of *HvAGP-S*, *HvSBE1*, and *HvSS2a* via yeast one-hybrid assay. Statistically significant differences are indicated: *, *P* < 0.05; **, *P* < 0.01 (Student’s *t*-test).

We compared all the members of the *HvAP2/ERF* family with the Barley Reference Transcript (BaRTv1.0) Dataset to check whether the identified genes were normally expressed in barley. All the identified *HvAP2/ERF* genes were matched to normal transcripts, thus indicating that the 185 genes functioned normally. The spatiotemporal expression profiles of *HvAP2/ERF* genes were analyzed in different tissues of barley using the published RNA-seq database (Supplementary Table 3). The *HvAP2/ERF* genes were expressed in at least one organ and were divided into 17 groups according to their expression patterns (Figure 5). Many genes were expressed in most tissues, although some were expressed only in a specific tissue. Among the latter, genes from the first, second, and 16th groups were more expressed at the middle stage of grain development. We selected some genes with high expression levels during grain development stages (5–25 DPA) for real-time qPCR analysis (Figure 6B). We also analyzed genes coding for starch biosynthesis-related enzymes (Figure 6A), namely, *HvAGP-L, HvAGP-S, HvWaxy, HvISA1, HvSS1, HvSS2a, HvSS3, HvSBE2a*, and *HvSBE2b*. The expression patterns clearly showed that all selected genes expressed differently from those of starch synthetase genes.

### *HvAP2-18* Bound Specifically to the Promoters of *HvAGP-S* and *HvSBE1* in LUC and Y1H Assays

We performed LUC and Y1H assays to further analyze a few selected TFs related to starch synthase genes. The NCBI-BLAST search revealed that *HvAP2-18* is a homologous gene of *RSR1*, which was previously identified in rice as a negative regulator of starch synthesis (Fu and Xue, 2010). However, the mechanisms activated by RSR1 to negatively regulate rice starch synthesis have not been thoroughly studied. LUC assay showed that HvAP2-18 bound the cis-acting DNA element in the promoter of the starch synthetase genes *AGP-S and SBE1* (Figure 6C). We use the Y1H system to confirm these interactions. The sequence of the starch synthase gene promoter region was constructed into the Y1H bait pABAi vector, and the candidate *HVAP2-18* was cloned into the Y1H ingruna carrier PGADT7 vector. The Y1H results confirmed that HvAP2-18 binds to the promoter region of the starch synthetases *AGP-S and SBE1*, indicating that *HvAP2-18* might be involved in the regulation of the starch synthesis in grain.

## Discussion

Starch accumulation occurs in barley endosperm and requires the coordinated regulation of various genes. Many TFs involved in starch production have been identified. The AP2/ERF family of TFs has a crucial role in regulating starch synthesis, as was identified in rice (Fu and Xue, 2010) and wheat. However, no member of the AP2/ERF family has been reported to regulate starch synthesis in barley. The accurate identification and analysis of genes from the *AP2/ERF* family might provide a reliable basis for the subsequent screening of candidate *HvAP2/ERF* genes involved in the regulation of starch synthesis. Previous work reported 121 *HvAP2/ERF* genes in barley (Guo et al., 2016). This study used an earlier version of the barley genome (Beier et al., 2017), which contains some gaps in the physical map and might therefore be lacking some important genes. The updated version (Morex V2) (Monat et al., 2019) of the barley genome has been recently released and is better than the earlier V1 annotation as it contains inclusive and broader information. Thus, the newest V2 version might be helpful for the precise characterization of the *Hv*AP2/ERF family (Monat et al., 2019). In the present study, a total of 185 *HvAP2/ERF* genes were identified in barley. Hence, we identified 64 more genes than the study using the V1 barley genome, confirming that the Morex V2 dataset contains more gene sequences with better annotation. The *HvERF3.1* and *HvERF3.2* identified previously matched a single gene, *HvERF-16*, in Morex V2 and Golden Promise genome databases. Similarly, the previously characterized *HvERF4.9* and *HvERF4.10* genes corresponded to *HvERF27*, and the *HvERF2.15*, *HvERF2.16*, and *HvERF2.17* genes matched the *HvERF-58* gene in the Morex V2 genome. These discrepancies might be caused by incorrect sequence splicing or misannotation in the earlier V1 genome. Moreover, multiple sequence alignments revealed that the results of the previous study included repeated genes. Thus, our results provide a more complete understanding of *HvAP2/ERF* gene family.

The *AP2/ERF* superfamily is a large and significant TF group and plays a role in various processes related to plant growth and development. It has been shown to be involved at different growth stages including seed germination, flowering and ripening as well as in response to various environmental stresses (Schmidt et al., 2013). With the advancement of second-generation sequencing technology, a lot of reports have identified *AP2/ERF* family members in various plant species (Nakano et al., 2006; Zhu et al., 2014; Zhao et al., 2019). The conserved motifs of AP2/ERF TFs have a specific role in the proper function of these genes (Sakuma et al., 2002). A total of 50 conserved motifs located outside the AP2 domain were detected in Arabidopsis (Nakano et al., 2006). Here, we analyzed 10 motifs in HvAP2/ERF proteins, and motif 1 (partial AP2/ERF domain) was observed in all genes (Figures 2, 3). All the AP2/ERF subfamilies contain the WLG domain except the AP2 subfamily, which has been confirmed by our study. Additionally, in the ERF subfamily, there was no WLG domain from *HvERF-38* to *Hv-ERF54*, but we still classified them into the ERF subfamily according to the annotation information (Supplementary Figure 2). Furthermore, we verified this classification via phylogenetic analysis. The genes *HvERF-38* to *HvERF-54* were part of the C1 group of the *ERF* subfamily because of their high homology (Figure 1) and the identical motif structures (Figure 3), confirming that they belong to the *ERF* subfamily.

Expression profiling has further confirmed that the AP2/ERF family has specific roles in various processes ranging from seed germination to fruit ripening and from response to environmental stress to response to pathogen attack (Klucher et al., 1996). However, there are relatively few studies on the transcriptional regulation of *AP2/ERF* genes during grain development. Transcriptomic expression analysis indicated that *HvAP2/ERF* genes were active in different barley tissues and showed tissue-specific differential expression. Most of the *HvAP2/ERF* genes of the first, second, and 16th groups were highly expressed in early grain developmental stages (5 DPA), but their expression levels were significantly decreased at 15 DPA. In the AP2 subfamily, 11 genes were highly expressed during endosperm development, with expression levels higher at 5 DPA compared with that at the 15 DPA. The other three subfamilies, namely, *ERF*, *RAV*, and *DREB*, contained 28, 1, and 26 genes, respectively, with higher expression levels during endosperm development. Although the relevant TFs involved in starch synthesis have not been identified in the study of the barley HvAP2/ERF family, it is possible to identify some family members involved in seed development and starch synthesis through the analysis of transcriptome data.

Previous work found that AP2/ERF TFs can regulate the expression of genes involved in starch synthesis. For example, *RSR1* was identified using gene co-expression analysis in rice (Fu and Xue, 2010) and wheat (Kang et al., 2013). Our results indicated that 12 *HvAP2/ERF* genes had an expression pattern similar to that of the starch synthesis genes (Figure 6). Among the starch synthesis enzymes, AGPase is responsible for the first key step of starch synthesis and is the rate-limiting enzyme (Ballicora et al., 2004). AGPase is a heterotetramer composed of two large subunits (AGPL) and two small subunits (AGP-S) in higher plants. AGPL and AGP-S have complementary roles in AGPase function (Qu et al., 2018). In wheat, *TubZIP28* and *TabZIP28* regulate starch synthesis by binding to the promoter of cytosolic AGPase and enhancing its transcription and activity (Song et al., 2020). *TaNAC-019-A1* regulates the expression of multiple starch synthase genes such as *AGP-S*, *SBE1*, and *SBE2a*, thus affecting starch synthesis in grains (Liu et al., 2020). In our study, LUC and Y1H analyses showed that *HvAP2-18* could bind to the promoter of *HvAGP-S* and *SBE1*. Additionally, *HvAP2-18* is homologous to the rice *RSR1* gene. The *RSR1* gene negatively regulates starch synthesis in endosperm. However, the mechanisms triggered by RSR1 to regulate starch synthesis in barley have not been studied in detail. We provide qRT-PCR data suggesting that the expression pattern of *HvAP2-18* was different to that of genes involved in starch synthesis. It was further observed by LUC and Y1H that *HvAP2-18* binds to the promoter region of starch synthesis genes. Additionally, transcriptome data and qPCR analyses showed that most *AP2* subfamily members, highly expressed at the grain development stage, tended to be highly expressed at the initial stage, whereas the expression was low later on. This pattern of expression suggests a possible negative regulatory role. The candidate gene *HvAP2-18* might therefore be the first transcriptional regulator of starch synthesis identified in barley and as such, could be a valuable target for further study.

## Data Availability Statement

The original contributions presented in the study are included in the article/Supplementary Material, further inquiries can be directed to the corresponding author/s.

## Author Contributions

JD carried out analysis of gene members, qRT-PCR and wrote this article. HK and YL downloaded sequences and did the yeast one-hybrid assay. WH, CG and NL analyzed the expression data. HT contributed to the material planting. QJ conceived and designed the experiments. QX, YZZ, PQ, YJ, MD, JM, JW, GC, XL, YW, and YLZ partially participated in its design and revised the manuscript. All authors read and approved the final manuscript.

## Conflict of Interest

The authors declare that the research was conducted in the absence of any commercial or financial relationships that could be construed as a potential conflict of interest.

## Publisher’s Note

All claims expressed in this article are solely those of the authors and do not necessarily represent those of their affiliated organizations, or those of the publisher, the editors and the reviewers. Any product that may be evaluated in this article, or claim that may be made by its manufacturer, is not guaranteed or endorsed by the publisher.

## Footnotes

1
http://webblast.ipk-gatersleben.de/barley_ibsc/

2
https://doi.ipk-gatersleben.de/DOI/83e8e186-dc4b-47f7-a820-28ad37cb176b/64687610-b20c-4702-9698-7dc2401a80e5/0

3
http://planttfdb.gao-lab.org

4
http://www.ncbi.nlm.nih.gov/Structure/cdd/cdd.shtml

5
https://galaxy-web.ipk-gatersleben.de/

6
https://galaxy-web.ipk-gatersleben.de/

7
http://ics.hutton.ac.uk/gmapper/index.html

8
http://www.megasoftware.net/

9
http://meme-suite.org/tools/meme

10
http://ics.hutton.ac.uk/barleyrtd/blast_page.html

11
http://ics.hutton.ac.uk/barleyrtd/about_material.html

## Supplementary Material

Supplementary Figure 1 Sequence alignment of proteins from different subfamilies of the *HvAP2/ERF* family.

Supplementary Figure 2 Conserved motifs of the 185 *HvAP2/ERF* genes.

Supplementary Figure 3 Gene structures of 185 barley *HvAP2/ERF* genes with full-length coding sequences.

## References

[B1] Bahaji A. Li J. Sánchez-López ÁM. Baroja-Fernández E. Muñoz F. J. Ovecka M. (2014). Starch biosynthesis, its regulation and biotechnological approaches to improve crop yields. *Biotechnol. Adv.* 32 87–106. 10.1016/j.biotechadv.2013.06.006 23827783

[B2] Bai A.-N. Lu X.-D. Li D.-Q. Liu J.-X. Liu C.-M. (2016). NF-YB1-regulated expression of sucrose transporters in aleurone facilitates sugar loading to rice endosperm. *Cell Res.* 26 384–388. 10.1038/cr.2015.116 26403192 PMC4783462

[B3] Ballicora M. A. Iglesias A. A. Preiss J. J. (2004). ADP-glucose pyrophosphorylase: a regulatory enzyme for plant starch synthesis. *Photosynth Res.* 79 1–24. 10.1023/b:pres.0000011916.67519.58 16228397

[B4] Beier S. Himmelbach A. Colmsee C. Zhang X.-Q. Barrero R. A. Zhang Q. (2017). Construction of a map-based reference genome sequence for barley, *Hordeum vulgare* L. *Sci. Data* 4:170044. 10.1038/sdata.2017.44 28448065 PMC5407242

[B5] Bello B. K. Hou Y. Zhao J. Jiao G. Wu Y. Li Z. (2019). NF-YB 1-YC 12-bHLH 144 complex directly activates Wx to regulate grain quality in rice (*Oryza sativa* L.). *Plant Biotechnol. J.* 17 1222–1235. 10.1111/pbi.13048 30552799 PMC6576074

[B6] Boutilier K. Offringa R. Sharma V. K. Kieft H. Ouellet T. Zhang L. (2002). Ectopic expression of BABY BOOM triggers a conversion from vegetative to embryonic growth. *Plant Cell* 14 1737–1749. 10.1105/tpc.001941 12172019 PMC151462

[B7] Chen C. Chen H. Zhang Y. Thomas H. R. Frank M. H. He Y. (2020). TBtools: an integrative toolkit developed for interactive analyses of big biological data. *Mol. Plant* 13 1194–1202. 10.1016/j.molp.2020.06.009 32585190

[B8] Chen J. Yi Q. Cao Y. Wei B. Zheng L. Xiao Q. (2016). ZmbZIP91 regulates expression of starch synthesis-related genes by binding to ACTCAT elements in their promoters. *J. Exp. Bot.* 67 1327–1338. 10.1093/jxb/erv527 26689855

[B9] Chuck G. Meeley R. B. Hake S. J. (1998). The control of maize spikelet meristem fate by theAPETALA2-like gene indeterminate spikelet1. *Genes Dev.* 12 1145–1154. 10.1101/gad.12.8.1145 9553044 PMC316712

[B10] Dong Q. Xu Q. Kong J. Peng X. Zhou W. Chen L. (2019). Overexpression of ZmbZIP22 gene alters endosperm starch content and composition in maize and rice. *Plant Sci.* 283 407–415. 10.1016/j.plantsci.2019.03.001 31128711

[B11] Elliott R. C. Betzner A. S. Huttner E. Oakes M. P. Tucker W. Gerentes D. (1996). AINTEGUMENTA, an APETALA2-like gene of *Arabidopsis* with pleiotropic roles in ovule development and floral organ growth. *Plant Cell* 8 155–168. 10.1105/tpc.8.2.155 8742707 PMC161088

[B12] Feng F. Qi W. Lv Y. Yan S. Xu L. Yang W. (2018). OPAQUE11 is a central hub of the regulatory network for maize endosperm development and nutrient metabolism. *Plant Cell* 30 375–396. 10.1105/tpc.17.00616 29436476 PMC5868688

[B13] Fu F.-F. Xue H.-W. (2010). Coexpression analysis identifies rice starch regulator1, a rice AP2/EREBP family transcription factor, as a novel rice starch biosynthesis regulator. *Plant Physiol.* 154 927–938. 10.1104/pp.110.159517 20713616 PMC2949045

[B14] Guo B. Wei Y. Xu R. Lin S. Luan H. Lv C. (2016). Genome-wide analysis of APETALA2/ethylene-responsive factor (AP2/ERF) gene family in barley (*Hordeum vulgare* L.). *PLoS One* 11:e0161322. 10.1371/journal.pone.0161322 27598245 PMC5012588

[B15] Hannah L. C. James M. (2008). The complexities of starch biosynthesis in cereal endosperms. *Curr. Opin. Biotechnol.* 19 160–165. 10.1016/j.copbio.2008.02.013 18400487

[B16] Huang H. Xie S. Xiao Q. Wei B. Zheng L. Wang Y. (2016). Sucrose and ABA regulate starch biosynthesis in maize through a novel transcription factor, ZmEREB156. *Sci. Rep.* 6:27590. 10.1038/srep27590 27282997 PMC4901336

[B17] James M. G. Denyer K. Myers A. M. (2003). Starch synthesis in the cereal endosperm. *Curr. Opin. Plant Biol.* 6 215–222. 10.1016/s1369-5266(03)00042-6 12753970

[B18] Jeon J.-S. Ryoo N. Hahn T.-R. Walia H. Nakamura Y. (2010). Starch biosynthesis in cereal endosperm. *Plant Physiol. Biochem.* 48 383–392. 10.1016/j.plaphy.2010.03.006 20400324

[B19] Jofuku K. D. Den Boer B. Van Montagu M. Okamuro J. K. (1994). Control of *Arabidopsis* flower and seed development by the homeotic gene APETALA2. *Plant Cell* 6 1211–1225. 10.1105/tpc.6.9.1211 7919989 PMC160514

[B20] Kang G.-Z. Xu W. Liu G.-Q. Peng X.-Q. Guo T.-C. (2013). Comprehensive analysis of the transcription of starch synthesis genes and the transcription factor RSR1 in wheat (*Triticum aestivum*) endosperm. *Genome* 56 115–122. 10.1139/gen-2012-0146 23517321

[B21] Keeling P. L. Myers A. M. (2010). Biochemistry and genetics of starch synthesis. *Annu. Rev. Food Sci. Technol.* 1 271–303. 10.1146/annurev.food.102308.124214 22129338

[B22] Klucher K. M. Chow H. Reiser L. Fischer R. L. (1996). The AINTEGUMENTA gene of *Arabidopsis* required for ovule and female gametophyte development is related to the floral homeotic gene APETALA2. *Plant Cell* 8 137–153. 10.1105/tpc.8.2.137 8742706 PMC161087

[B23] Kumar R. Taware R. Gaur V. S. Guru S. Kumar A. J. (2009). Influence of nitrogen on the expression of TaDof1 transcription factor in wheat and its relationship with photo synthetic and ammonium assimilating efficiency. *Mol. Biol. Rep.* 36 2209–2220. 10.1007/s11033-008-9436-8 19123069

[B24] Lata C. Mishra A. K. Muthamilarasan M. Bonthala V. S. Khan Y. Prasad M. J. (2014). Genome-wide investigation and expression profiling of AP2/ERF transcription factor superfamily in foxtail millet (Setaria italica L.). *PLoS One* 9:e113092. 10.1371/journal.pone.0113092 25409524 PMC4237383

[B25] Liu G. Wu Y. Xu M. Gao T. Wang P. Wang L. (2016). Virus-induced gene silencing identifies an important role of the TaRSR1 transcription factor in starch synthesis in bread wheat. *Int. J. Mol. Sci.* 17:1557. 10.3390/ijms17101557 27669224 PMC5085620

[B26] Liu J. Chen N. Chen F. Cai B. Dal Santo S. Tornielli G. B. (2014). Genome-wide analysis and expression profile of the bZIP transcription factor gene family in grapevine (*Vitis vinifera*). *BMC Genomics* 15:281. 10.1186/1471-2164-15-281 24725365 PMC4023599

[B27] Liu S. Wang X. Wang H. Xin H. Yang X. Yan J. (2013). Genome-wide analysis of ZmDREB genes and their association with natural variation in drought tolerance at seedling stage of *Zea mays* L. *PLoS Genet* 9:e1003790. 10.1371/journal.pgen.1003790 24086146 PMC3784558

[B28] Liu Y. Hou J. Wang X. Li T. Majeed U. Hao C. (2020). The NAC transcription factor NAC019-A1 is a negative regulator of starch synthesis in wheat developing endosperm. *J. Exp. Bot.* 71 5794–5807. 10.1093/jxb/eraa333 32803271

[B29] Ma J. Yang Y. Luo W. Yang C. Ding P. Liu Y. (2017). Genome-wide identification and analysis of the MADS-box gene family in bread wheat (*Triticum aestivum* L.). *PLoS One* 12:e0181443. 10.1371/journal.pone.0181443 28742823 PMC5526560

[B30] Marchler-Bauer A. Lu S. Anderson J. B. Chitsaz F. Derbyshire M. K. Deweese-Scott C. (2010). CDD: a conserved domain database for the functional annotation of proteins. *Nucleic Acids Res.* 39 D225–D229. 21109532 10.1093/nar/gkq1189 PMC3013737

[B31] Mascher M. Gundlach H. Himmelbach A. Beier S. Twardziok S. O. Wicker T. (2017). A chromosome conformation capture ordered sequence of the barley genome. *Nature* 544 427–433. 28447635 10.1038/nature22043

[B32] Mayer K. Waugh R. Langridge P. Close T. Wise R. Graner A. (2012). A physical, genetic and functional sequence assembly of the barley genome. *Nature* 491 711–716. 10.1038/nature11543 23075845

[B33] Monat C. Padmarasu S. Lux T. Wicker T. Gundlach H. Himmelbach A. (2019). TRITEX: chromosome-scale sequence assembly of *Triticeae* genomes with open-source tools. *Genome Biol.* 20:284. 10.1186/s13059-019-1899-5 31849336 PMC6918601

[B34] Moose S. P. Sisco P. H. (1996). Glossy15, an APETALA2-like gene from maize that regulates leaf epidermal cell identity. *Genes Dev.* 10 3018–3027. 10.1101/gad.10.23.3018 8957002

[B35] Nakamura T. Yamamori M. Hirano H. Hidaka S. Nagamine T. J. M. Mgg G. G. (1995). Production of waxy (amylose-free) wheats. *Mol. Gen. Genet.* 248 253–259. 10.1007/BF02191591 7565586

[B36] Nakano T. Suzuki K. Fujimura T. Shinshi H. J. (2006). Genome-wide analysis of the ERF gene family in *Arabidopsis* and rice. *Plant Physiol.* 140 411–432. 10.1104/pp.105.073783 16407444 PMC1361313

[B37] Niu B. Deng H. Li T. Sharma S. Yun Q. Li Q. (2020). OsbZIP76 interacts with OsNF-YBs and regulates endosperm cellularization in rice (*Oryza sativa*). *J. Int. Plant Biol.* 62 1983–1996. 10.1111/jipb.12989 32621654

[B38] Pérez L. Soto E. Farré G. Juanos J. Villorbina G. Bassie L. (2019). CRISPR/Cas9 mutations in the rice Waxy/GBSSI gene induce allele-specific and zygosity-dependent feedback effects on endosperm starch biosynthesis. *Plant Cell Rep.* 38 417–433. 10.1007/s00299-019-02388-z 30715580

[B39] Qu J. Xu S. Zhang Z. Chen G. Zhong Y. Liu L. (2018). Evolutionary, structural and expression analysis of core genes involved in starch synthesis. *Sci. Rep.* 8:12736. 10.1038/s41598-018-30411-y 30143668 PMC6109180

[B40] Rapazote-Flores P. Bayer M. Milne L. Mayer C.-D. Fuller J. Guo W. (2019). BaRTv1. 0: an improved barley reference transcript dataset to determine accurate changes in the barley transcriptome using RNA-seq. *BMC Genomics* 20:968. 10.1186/s12864-019-6243-7 31829136 PMC6907147

[B41] Riechmann J. L. Meyerowitz E. M. (1998). The AP2/EREBP family of plant transcription factors. *Biol. Chem.* 379 633–646. 9687012 10.1515/bchm.1998.379.6.633

[B42] Sakuma Y. Liu Q. Dubouzet J. G. Abe H. Shinozaki K. Yamaguchi-Shinozaki K. J. B. (2002). DNA-binding specificity of the ERF/AP2 domain of Arabidopsis DREBs, transcription factors involved in dehydration-and cold-inducible gene expression. *Biochem. Biophys. Res. Commun.* 290 998–1009. 10.1006/bbrc.2001.6299 11798174

[B43] Schmidt R. Mieulet D. Hubberten H.-M. Obata T. Hoefgen R. Fernie A. R. (2013). SALT-RESPONSIVE ERF1 regulates reactive oxygen species–dependent signaling during the initial response to salt stress in rice. *Plant Cell* 25 2115–2131. 10.1105/tpc.113.113068 23800963 PMC3723616

[B44] Song Y. Luo G. Shen L. Yu K. Yang W. Li X. (2020). TubZIP28, a novel bZIP family transcription factor from *Triticum urartu*, and TabZIP28, its homologue from *Triticum aestivum*, enhance starch synthesis in wheat. *New Phytol.* 226 1384–1398. 10.1111/nph.16435 31955424

[B45] Sonnewald U. Kossmann J. J. (2013). Starches—from current models to genetic engineering. *Plant Biotechnol. J.* 11 223–232. 10.1111/pbi.12029 23190212

[B46] Sun C. Palmqvist S. Olsson H. Borén M. Ahlandsberg S. Jansson C. J. (2003). A novel WRKY transcription factor, SUSIBA2, participates in sugar signaling in barley by binding to the sugar-responsive elements of the iso1 promoter. *Plant Cell* 15 2076–2092. 10.1105/tpc.014597 12953112 PMC181332

[B47] Wang J.-C. Xu H. Zhu Y. Liu Q.-Q. Cai X.-L. J. (2013). OsbZIP58, a basic leucine zipper transcription factor, regulates starch biosynthesis in rice endosperm. *J. Exp. Bot.* 64 3453–3466. 10.1093/jxb/ert187 23846875 PMC3733163

[B48] Yamaguchi-Shinozaki K. Shinozaki K. J. (1994). A novel cis-acting element in an *Arabidopsis* gene is involved in responsiveness to drought, low-temperature, or high-salt stress. *Plant Cell* 6 251–264. 8148648 10.1105/tpc.6.2.251 PMC160431

[B49] Zhang G. Chen M. Chen X. Xu Z. Guan S. Li L.-C. (2008). Phylogeny, gene structures, and expression patterns of the ERF gene family in soybean (*Glycine max* L.). *J. Exp. Bot.* 59 4095–4107. 10.1093/jxb/ern248 18832187 PMC2639015

[B50] Zhang J. Chen J. Yi Q. Hu Y. Liu H. Liu Y. (2014). Novel role of ZmaNAC36 in co-expression of starch synthetic genes in maize endosperm. *Plant Mol. Biol.* 84 359–369. 10.1007/s11103-013-0153-x 24235061

[B51] Zhang Z. Dong J. Ji C. Wu Y. Messing J. (2019). NAC-type transcription factors regulate accumulation of starch and protein in maize seeds. *PNAS* 116 11223–11228. 10.1073/pnas.1904995116 31110006 PMC6561305

[B52] Zhang Z. Zheng X. Yang J. Messing J. Wu Y. (2016). Maize endosperm-specific transcription factors O2 and PBF network the regulation of protein and starch synthesis. *Proc. Natl. Acad. Sci. U S A.* 113 10842–10847. 10.1073/pnas.1613721113 27621432 PMC5047157

[B53] Zhao Y. Ma R. Xu D. Bi H. Xia Z. Peng H. (2019). Genome-wide identification and analysis of the AP2 transcription factor gene family in wheat (*Triticum aestivum* L.). *Front. Plant Sci.* 10:1286. 10.3389/fpls.2019.01286 31681381 PMC6797823

[B54] Zhou J. Tang X. Martin G. B. (1997). The Pto kinase conferring resistance to tomato bacterial speck disease interacts with proteins that bind a cis-element of pathogenesis-related genes. *EMBO J.* 16 3207–3218. 10.1093/emboj/16.11.3207 9214637 PMC1169938

[B55] Zhu X. Qi L. Liu X. Cai S. Xu H. Huang R. (2014). The wheat ethylene response factor transcription factor pathogen-induced ERF1 mediates host responses to both the necrotrophic pathogen *Rhizoctonia cerealis* and freezing stresses. *Plant Physiol.* 164 1499–1514. 10.1104/pp.113.229575 24424323 PMC3938636

