# Supplementary figures and images for: Re-examination of the APETALA2/Ethylene-Responsive Factor Gene Family in Barley (Hordeum vulgare L.) Indicates a Role in the Regulation of Starch Synthesis
Source: Front Plant Sci. 2021 Dec 1;12:791584. doi: 10.3389/fpls.2021.791584 (PMC8672199; doi:10.3389/fpls.2021.791584)

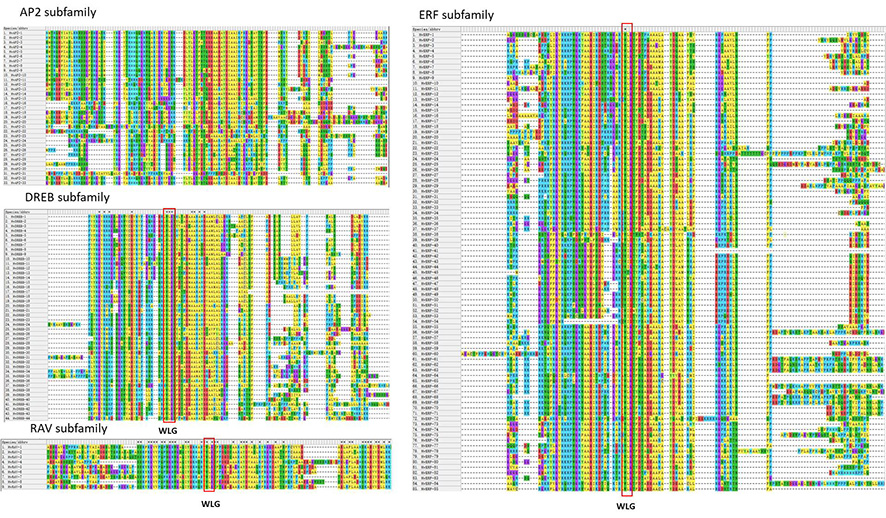

Supplement: Supplementary Figure 1 — Sequence alignment of proteins from different subfamilies of the HvAP2/ERF family. [file Image_1.JPEG]

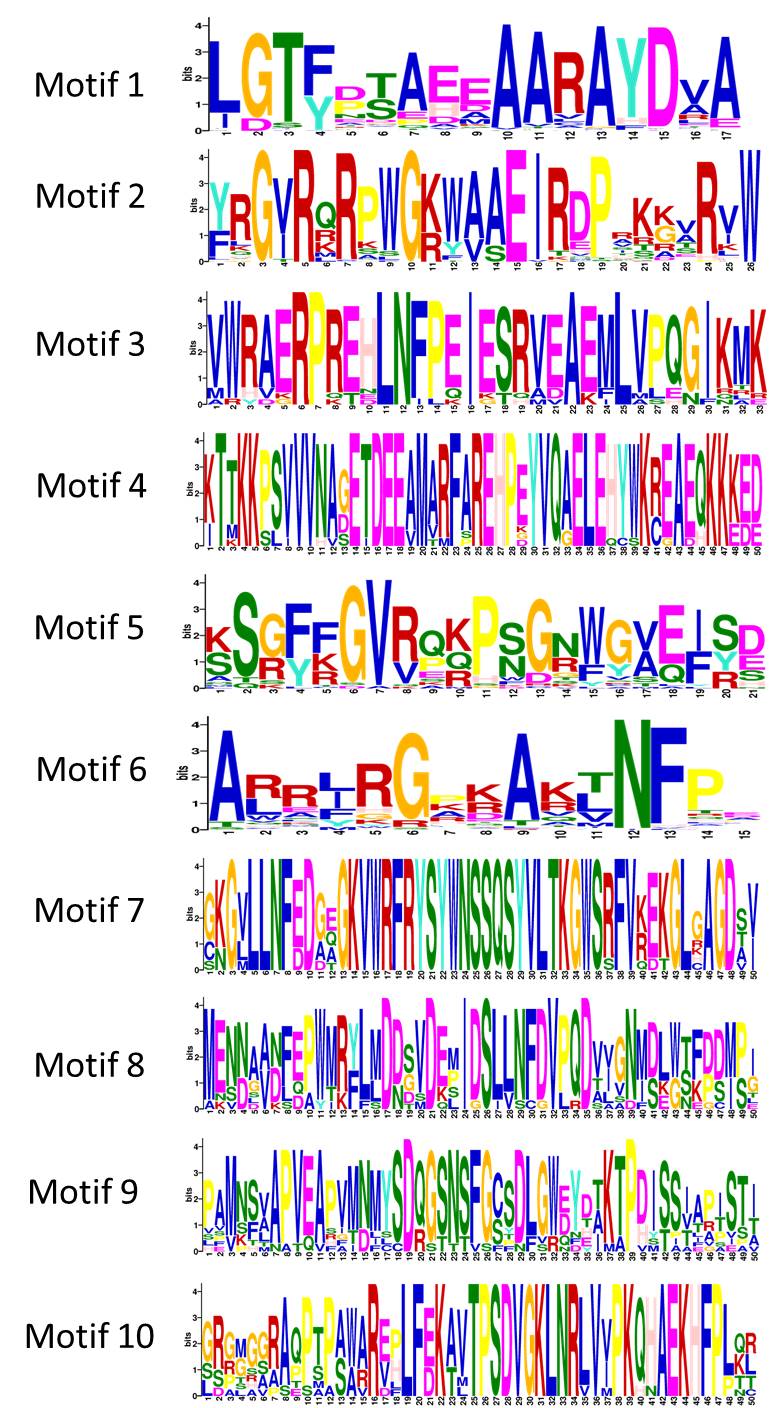

Supplement: Supplementary Figure 2 — Conserved motifs of the 185 HvAP2/ERF genes. [file Image_2.JPEG]

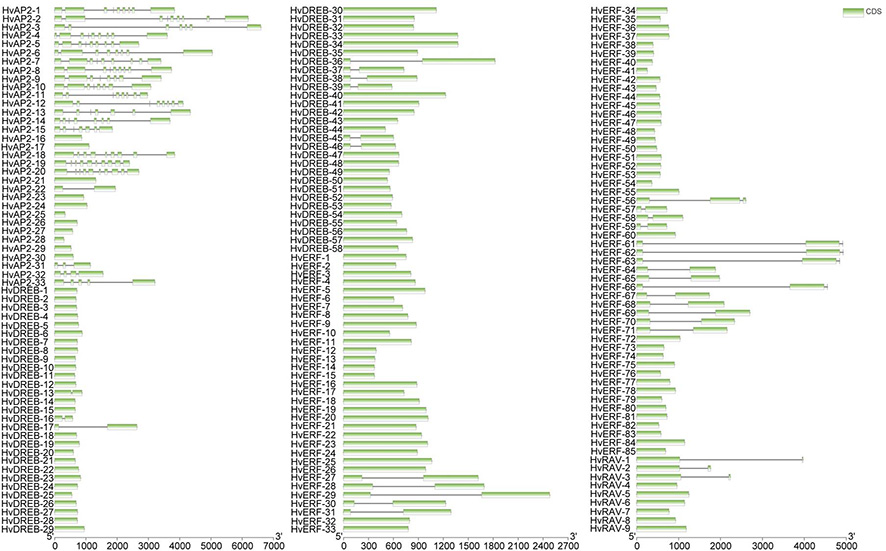

Supplement: Supplementary Figure 3 — Gene structures of 185 barley HvAP2/ERF genes with full-length coding sequences. [file Image_3.JPEG]
